# In pursuit of excellence for patients with cancer: the Scottish Cancer Therapy Network model

**DOI:** 10.1038/sj.bjc.6690262

**Published:** 1999-04

**Authors:** P L Stroner, D H Brewster, J A Dewar, O Eremin, A Gould, G C Howard, S B Kaye

**Affiliations:** Information & Statistics Division, National Health Service in Scotland, Information and Statistics Division, Room C020, Trinity Park House, South Trinity Road, Edinburgh, EH5 3SQ, UK; Ninewells Hospital and Medical School, Dundee, DD1 9SY, UK; University of Aberdeen Faculty of Medicine, Aberdeen, AB9 2ZD, UK; Western General Hospital, Edinburgh, EH4 2XU, UK; Beatson Oncology Centre, Glasgow, G11 6NT, UK

**Keywords:** clinical trials, information services, medical audit, neoplasms, practice guidelines, Scotland

## Abstract

The Scottish Cancer Therapy Network (SCTN) was created against a background of rising concerns about perceived variation in the quality of care available to patients with cancer. SCTN has established itself as a major organization with the necessary recognition and infrastructure to provide leadership, support and impetus in the field of clinical guidelines, clinical audit and clinical trials of cancer therapy in Scotland. Since being formed in 1993, SCTN has been instrumental in the development of three evidence-based, clinical guidelines and in the completion of detailed, national, retrospective audits of the treatment of five major tumour sites. The infrastructure has been used successfully to support and encourage trial participation. Challenges for the future are a re-orientation towards prospective audit, widening the constituency and sense of ownership of SCTN as a resource for practising clinicians, and further increasing recruitment into clinical trials. © 1999 Cancer Research Campaign


*British Journal of Cancer* (1999) **79**(11/12), 1641–1645
© 1999 Cancer Research Campaign
Article no. bjoc.1998.0262

Review

### In pursuit of excellence for patients with cancer: the Scottish Cancer Therapy Network model

**PL Stroner[1], DH Brewster[1], JA Dewar[2], O Eremin[3], A Gould[1], GC Howard[4] and SB Kaye[5]**

[1]Information & Statistics Division, National Health Service in Scotland, Room C020, Information and Statistics Division, Trinity Park House, South Trinity Road, Edinburgh EH5 3SQ, UK; [2]Ninewells Hospital and Medical School, Dundee DD1 9SY, UK; [3]University of Aberdeen Faculty of Medicine, Aberdeen AB9 2ZD, UK; [4]Western General Hospital, Edinburgh EH4 2XU, UK; [5]Beatson Oncology Centre, Glasgow G11 6NT, UK

**Summary** The Scottish Cancer Therapy Network (SCTN) was created against a background of rising concerns about perceived variation in the quality of care available to patients with cancer. SCTN has established itself as a major organization with the necessary recognition and infrastructure to provide leadership, support and impetus in the field of clinical guidelines, clinical audit and clinical trials of cancer therapy in Scotland. Since being formed in 1993, SCTN has been instrumental in the development of three evidence-based, clinical guidelines and in the completion of detailed, national, retrospective audits of the treatment of five major tumour sites. The infrastructure has been used successfully to support and encourage trial participation. Challenges for the future are a re-orientation towards prospective audit, widening the constituency and sense of ownership of SCTN as a resource for practising clinicians, and further increasing recruitment into clinical trials.

**Keywords:** clinical trials; information services; medical audit; neoplasms; practice guidelines; Scotland

Cancer is a major cause of morbidity and mortality in the Scottish population (Information & Statistics Division, 1998). In recent years, there has been rising public and political concern about perceived variation in the quality of care for patients with cancer in the UK. Although there are difficulties in interpretation, the results of the EUROCARE study suggest that the survival prospects for patients with cancer in the UK may be less favourable than those reported by some other European countries (Berrino et al, 1995). A number of recently published studies on historical cohorts of patients with cancer in Scotland have reported variations in outcomes apparently related to patterns of care (McArdle and Hole, 1991, 1996; Junor et al, 1994; Gillis and Hole, 1996).

### FORMATION OF THE SCOTTISH CANCER THERAPY NETWORK

The Scottish Cancer Therapy Network (SCTN) was established in early 1993 with a 5-year grant of £250 000 per annum from the Chief Scientist Office and the Clinical Resource and Audit Group, both of which were part of the then Scottish Office Home and Health Department The overall aim of the SCTN is to help ensure that optimal treatment is available throughout Scotland to all patients with cancer, by providing up-to-date information on clinical cancer trials, audits of outcome, best current practice for the treatment of cancer and patterns of cancer care throughout Scotland.

### Steering committee

A steering committee, relating closely to the already established Scottish Cancer Coordinating and Advisory Committee (SCCAC) (Aitken et al, 1994), was established to guide the work of the Network for the first 5 years. It was chaired by the Chief Scientist; membership included the Chief Medical Officer for Scotland, five SCTN grantholders, independent members with special expertise, and representatives of cancer charities, the Medical Research Council and the Scottish Breast Screening Programme.

### Grantholders

Until recently, the operational aspects of SCTN activities were guided by five grantholders. The grantholders were chosen to represent a central office and the catchment areas of the four academic cancer centres in Scotland, as well as the disciplines of cancer registration/epidemiology, surgical oncology, clinical oncology and medical oncology. The grantholders met every 8–12 weeks to monitor progress, debate strategy and priorities, and to discuss the problems and practicalities of implementation of SCTN activities.

### STRUCTURE AND STAFFING

The central theme of SCTN's work is that it covers the whole of Scotland. To this end, a central office and five regional offices have been established (Figure 1).

The central office is staffed by a coordinator (statistician), and computing, information and administration staff. Five study co-ordinators are supported by additional grants to work on specific projects. The central office is located adjacent to the Scottish Cancer Registry in the Information & Statistics Division of the Common Services Agency for the National Health Service (NHS) in Scotland. This allows SCTN to make effective use of centrally held information on cancer, including record linkage of mortality data, which facilitates analysis of survival outcome. The central office is responsible for co-ordination of projects, computer support, statistical analysis and dissemination of information. Personnel and financial services, including administration of the grant, are provided by the host organization.

*Received 16 June 1998*
*Accepted 20 August 1998*

*Correspondence to:* PL Stroner

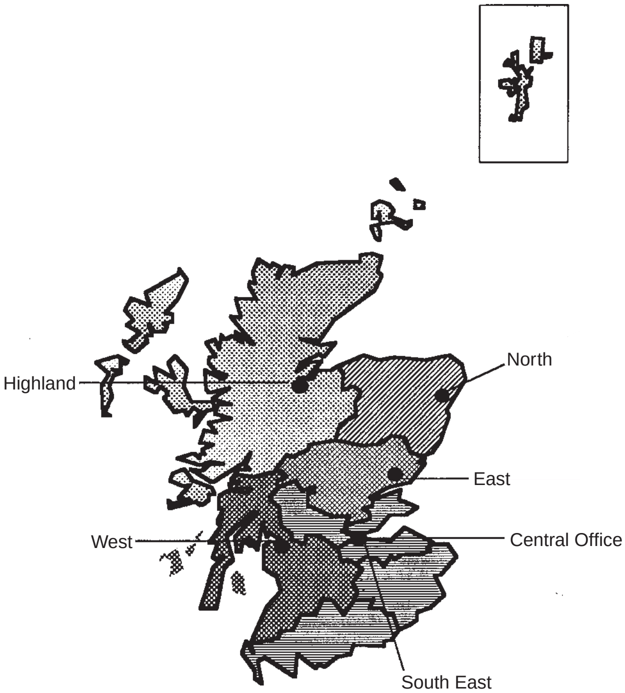

**Figure 1** Location of SCTN offices and staff

The regional and affiliated offices are staffed by data managers. They work closely with clinicians, providing administrative support for participation in clinical trials; they also collect data for national audits organized by SCTN.

### ACHIEVEMENTS

The work of SCTN can be grouped under four main headings: clinical guidelines; clinical audit; clinical trials and dissemination of information. Much of SCTN's work relating to breast cancer, colorectal cancer and lung cancer has been guided by three multidisciplinary, site-specific Focus Groups which were established with a defined remit (Box 1) under the joint auspices of SCCAC and the SCTN Steering Committee.

In a departure from this site-specific model, and in recognition of the importance of palliative care, a Palliative Care Focus Group was established in 1996. This group has been charged with tackling many of the 'generic' aspects of palliative care that are common across a range of cancer sites and types. SCTN central office staff provide the secretariat for each of the Focus Groups.

**Box 1** Remit of site-specific focus groups

'…to examine information about the tumour type from the point of view of aetiology, prevention (including education and screening), diagnosis, treatment and palliation; adequacy of existing arrangements and best practice should be considered under each of these headings. The group should also identify past, current and future research and audit studies which will inform practice.'

### Clinical guidelines

An early priority for SCTN was the development and, with others, the subsequent dissemination of multidisciplinary, evidence-based clinical guidelines relating to the major sites of cancer. Guidelines have been developed by the site-specific Focus Groups in accordance with the stringent criteria of the Scottish Intercollegiate Guidelines Network (SIGN) (SIGN, 1995) for colorectal cancer (SCTN/SIGN, 1997), lung cancer (SCTN/SIGN, 1998*a*), and breast cancer (SCTN/SIGN, 1998*b*). Each set of guidelines contains a minimum core dataset based on their key, measurable recommendations and designed to monitor their implementation nationally.

### Clinical audit

SCTN has conducted a series of national, retrospective audits of breast cancer (Scottish Breast Cancer Focus Group et al, 1996), colorectal cancer (analysis in progress) and lung cancer (analysis in progress). In addition, an audit of prostate cancer (Goodman, 1997) has been carried out in collaboration with the Scottish Urological Oncology Group, and an audit of ovarian cancer (Junor, 1997) has been carried out in collaboration with colleagues from the West of Scotland Cancer Surveillance Unit and Glasgow's Beatson Oncology Centre.

These audits aim to include all patients in Scotland diagnosed with the cancer in question during a given period. Patients were identified from the Scottish Cancer Registry which holds data believed to be of high quality, both in terms of accuracy (Brewster et al, 1994) and ascertainment (Brewster et al, 1996, 1997) (Table 1).

Patients were eligible for inclusion in the audits if their cancer registration diagnosis was valid, if they had not had a previous malignant neoplasm, and if they were diagnosed and treated in Scotland during the calendar year of the audit.

As well as providing information on recent practice (Boxes 2–4), the audit databases permit an assessment of the resource implications of recommendations in clinical guidelines and act as a baseline against which future changes in clinical practice can be measured. The data have also been used for the purpose of health

**Table 1** SCTN national retrospective audits: potential study populations, availability of medical records and status by cancer site

| Cancer site (Year(s) of diagnosis) | No. of cases registered | No. (%) of notes retrieved | Status |
|---|---|---|---|
| Breast (1987 & 1993) | 5471 | 5135 (93.8%) | Reported |
| Prostate (1988 & 1993) | 2812 | 2575 (91.6%) | Analysis in progress |
| Colorectal (1993) | 3243 | 3031 (93.5%) | Analysis in progress |
| Ovary (1992, 1993, 1994) | 1638 | 1505 (91.9%) | Analysis in progress |
| Lung (1995) | 4297 | 4049 (94.2%) | Analysis in progress |
| Total | 17 461 | 16 295 (93.3%) | |

© Cancer Research Campaign 1999

**Table 2** Recruitment of patients in Scotland into selected breast cancer trials

| Trial | Scotland | Rest of UK | % (Scotland of UK total) |
|---|---|---|---|
| ABC Premenopausal | 260 | 854 | 23 |
| ABC Postmenopausal | 248 | 395 | 39 |
| Anglo Celtic High Dose | 79 | 268 | 29 |
| ATAC | 193 | 1302 | 13 |
| UKDCIS | 223 | 1195 | 16 |
| BASOII | 183 | 509 | 26 |
| Total | 1186 | 4523 | 21 |

**Box 2** National Scottish Breast Cancer Audit 1987 and 1993 – Key results

The results showed that between 1987 and 1993, there was:

- an increase in the proportion referred to surgeons with a specialist interest (≥ 50 patients/year, or working in teams) from 35% to 57%.
- an increase in the proportion of patients having breast conservation surgery (40% to 52%) and postoperative radiotherapy (55% to 75%).
- an increase in the proportion having axillary surgery performed (79% to 90%)
- an increase in the use of systemic adjuvant therapy, including an increase in the proportion of premenopausal, node-positive patients receiving chemotherapy (35% to 75%).
- no increase in the proportion of patients having tumour oestrogen receptors measured.
- Survival analysis of the 1987 cohort showed that the only non-treatment factor to influence survival was Health Board of first treatment. Part of the variation by Health Board seemed to be explained by the variation in the proportion of patients managed non-surgically. There was also a suggestion that survival by Health Board was correlated with the use of systemic adjuvant therapy in 1987.
- The proportion of women entering clinical trials was 12% in 1987 and remained unchanged in 1993. Patients were more likely to be entered into a clinical trial if they were referred to an oncologist or seen by a surgeon with a large case load.

**Box 3** National Scottish Prostate Cancer Audit 1988 and 1993 – Key results

The following were the main important findings of the study:

- In both years, thorough tumour staging was often omitted or poorly documented.
- Bone scans were used in only 43% of patients for staging purposes in 1988, increasing to 58% by 1993.
- Use of radical radiotherapy showed marked regional variation; the use of radical surgery, while still infrequent, increased over the study period.
- There was a substantial decline in surgical methods of hormonal manipulation (29% to 12%).
- A surprisingly high use of stilboestrol was seen in 1988 (12%) with marked regional variation; some use persisted even into 1993 (1%).

service research to establish which patterns or features of care appear to be advantageous in terms of patient outcomes.

In collaboration with the South East Scotland Lung Cancer Group, SCTN has undertaken responsibility for coordination, administration and statistical analysis of a NHS Research and Development (R&D)-funded prospective audit of lung cancer management, with particular emphasis on quality of life and resource use. SCTN are also collaborating with palliative care colleagues throughout Scotland in a prospective audit of pain control and prescribing standards.

### Clinical trials

SCTN provides administration and coordination for a number of large multicentre, national and international studies. It works in a complementary and collaborative fashion to other trials offices in Scotland and the rest of the UK, and with the European Organisation for Research and Treatment of Cancer, in Brussels. In particular, SCTN works closely with, and provides the secretariat for, the Scottish Cancer Trials Breast Group (SCTBG). The collaboration with SCTBG has been successful and the level of recruitment into studies for which the group are responsible exceeds the expected per capita level of 10% of the UK total (Table 2). SCTN central office staff also provide statistical advice and administrative support to clinicians involved in the development of clinical trial protocols. The central office has developed a computerized clinical trials management system to facilitate randomization, data entry and form tracking.

SCTN is represented on several national trial steering groups and has built a reputation as a competent and professional trials office. Despite recent recognized barriers to clinical research (Smyth et al, 1994), the network of regional data managers has succeeded in raising awareness of existing trials and has provided assistance to clinicians throughout Scotland by facilitating applications to local and multicentre research ethics committees, trial entry and data collection. For example, in collaboration with the SCTBG, SCTN has assisted some cancer units which had not previously had the resources necessary to enter patients into clinical trials.

Again in collaboration with the SCTBG, SCTN has been very successful in attracting funding for trials in breast cancer. It is important to note that the results of all these trials, whether funded by charities, research councils or the pharmaceutical industry, will be in the public domain and are trials of combination therapies or management approaches rather than of specific drugs.

SCTN regional data managers provide assistance to clinicians participating in national trials, administered by other trials offices, such as the AXIS and QUASAR trials in colorectal cancer.

### Dissemination of information

Information is disseminated in a variety of ways. A quarterly *SCTN Newsletter* is now well-established with a multidisciplinary circulation in excess of 1000. Four annual reports have been

© Cancer Research Campaign 1999

**Box 4** National Scottish Ovarian Cancer Audit 1992–1994 – Key results

Comparing the findings for 1993 with those of a previous (1987) audit:

- There was no significant change in the distribution of stage, or of any of the other prognostic factors, between the 1987 and 1993 audits.
- There was a substantial increase in the proportion of women receiving platinum-based chemotherapy as well as an increase in those being managed by a multidisciplinary team.
- There was a move towards more patients receiving combination chemotherapy. In contrast, neither the proportion of women first seen by a gynaecologist, nor the number operated on by a gynaecologist, changed.
- The question raised by the changes in practice is whether these have led to an improvement in survival. Provisionally, survival has improved by 6% at 2 years for patients aged under 70 years.

produced and distributed amongst a wide range of disciplines. The results of the breast cancer audit have been published in a special report (Scottish Breast Cancer Focus Group et al, 1996) and publications are beginning to appear in peer-reviewed journals (Twelves et al, 1998*a*, 1998*b*). During their development, the clinical guidelines were presented and discussed at open meetings attended by a wide range of delegates at the Royal College of Physicians of Edinburgh.

The rapid changes in information technology and the exponential growth of the Internet present new opportunities for the dissemination of information to a wider audience. SCTN is currently developing a website in collaboration with their host organization.

### FUTURE PLANS

Against the background of a major re-organization of cancer services, following the Calman/Hine Report (Department of Health Expert Advisory Group on Cancer, 1995) and related reports in Scotland (SCCAC, 1996, 1997), SCTN faces a number of challenges in the future. Firstly, credible, contemporary data will be needed to monitor the impact of the re-organization of cancer services, and the dissemination and implementation of evidence-based clinical guidelines. Thus, it is necessary for SCTN to shift its emphasis away from retrospective audit towards the facilitation and support of local, but (with the support of the Scottish Cancer Registry) population-based, prospective audit. The second, related challenge, is for SCTN to extend its network further as a facilitating organization, particularly involving cancer units. Finally, it is important for SCTN to increase recruitment into clinical trials involving patients with lung and colorectal cancer, to match the success in breast cancer. This will be a new focus of attention for the Colorectal and Lung Cancer Focus Groups.

Following a review in June 1997, funding for SCTN has been secured for a further 3 years, in the first instance. The management arrangements for SCTN have altered to reflect changes in the structure of cancer committees within the Scottish Office Department of Health. SCCAC and the SCTN Steering Committee have ceased to exist and their functions have, in effect, been taken over by a newly formed Cancer Executive Group which will oversee the activities of SCTN and will report, through a newly formed Scottish Cancer Group, to the Chief Medical Officer for Scotland.

### CONCLUSION

Since its formation in 1993, SCTN has established itself as a major organization with the necessary recognition and infrastructure to provide leadership, support and impetus in the field of clinical guidelines, clinical audit and clinical trials of cancer therapy in Scotland. Challenges for the future are a re-orientation towards prospective audit, widening the constituency and sense of ownership of SCTN as a resource for practising clinicians, and increasing further recruitment into clinical trials.

### ACKNOWLEDGEMENTS

Dr Calum Muir (deceased) and Professor Bill Duncan were two of the original SCTN grantholders and were instrumental in the establishment of SCTN and in ensuring its success. Dr David Whillis oversees the activity of the affiliated office of SCTN which covers the territory of Highland and Western Isles Health Boards. SCTN would not be able to function without the goodwill, enthusiasm, support and commitment of many other clinical colleagues who give freely of their spare time. We are grateful to Willie Farquhar and Arthur White, both of the Scottish Office Department of Health, for their helpful comments on an earlier version of this manuscript.

SCTN is funded by grants from the Chief Scientist Office and the Clinical Resource and Audit Group, both of the Scottish Office Department of Health. However, the views expressed in this paper are those of the authors.

### REFERENCES

Aitken REG, Farquhar W and Moir ATB (1994) Cancer: advisory and co-ordinating bodies. *Health Bull (Edin)* **52**: 47–50

Berrino F, Sant M, Verdecchia A, Capocaccia R, Hakulinen T and Estève J (eds) (1995) *Survival of Cancer Patients in Europe (The EUROCARE Study)*. IARC Scientific Publications No 132. International Agency for Research on Cancer: Lyon

Brewster D, Crichton J and Muir CS (1994) How accurate are Scottish cancer registration data? *Br J Cancer* **70**: 954–960

Brewster D, Crichton J, Harvey JC, Dawson G and Nairn ER (1996) Benefits and limitations of pathology databases to cancer registries. *J Clin Pathol* **49**: 947–949

Brewster D, Crichton J, Harvey JC and Dawson G (1997) Completeness of case ascertainment in a Scottish Regional Cancer Registry for the year 1992. *Public Health* **111**: 339–343

Department of Health Expert Advisory Group on Cancer (1995) *A Policy Framework for Commissioning Cancer Services*. A Report by the Expert Advisory Group on Cancer to the Chief Medical Officers of England and Wales. Department of Health: London

Gillis CR and Hole DJ (1996) Survival outcome of care by specialist surgeons in breast cancer: a study of 3786 patients in the west of Scotland. *Br Med J* **312**: 145–148

Goodman CM on behalf of the Scottish Urological Oncology Group and Scottish Cancer Therapy Network (1997) Audit of prostate cancer management in Scotland: 1988 and 1993. *Br J Urol* **79** (Suppl 4): 7

Information & Statistics Division (1998). *Scottish Health Statistics 1997*. ISD Scotland: Edinburgh

Junor EJ, Hole DJ and Gillis CR (1994) Management of ovarian cancer: referral to a multidisciplinary team matters. *Br J Cancer* **70**: 363–370

Junor EJ on behalf of the Scottish Cancer Therapy Network (1997) Ovarian cancer: improvement in survival in Scotland with platinum chemotherapy. *Br J Cancer* **76** (Suppl 1): 15–16

McArdle CS and Hole D (1991) Impact of variability among surgeons on postoperative morbidity and mortality and ultimate survival. *Br Med J* **302**: 1501–1505

*© Cancer Research Campaign 1999*

McArdle CS and Hole D (1996) An analysis of outcome following gastric cancer surgery. *GI Cancer* **1**: 177–182

Scottish Breast Cancer Focus Group, Scottish Cancer Trials Breast Group, Scottish Cancer Therapy Network (1996) *Scottish Breast Cancer Audit 1987 & 1993*. SCTN: Edinburgh

Scottish Cancer Co-ordinating and Advisory Committee (1996) *Commissioning Cancer Services in Scotland*. Report to the Chief Medical Officer. Scottish Office Department of Health: Edinburgh

Scottish Cancer Co-ordinating and Advisory Committee (1997) *Commissioning Cancer Services in Scotland*. *Primary and Palliative Care Services*. Report to the Chief Medical Officer. Scottish Office Department of Health: Edinburgh

Scottish Cancer Therapy Network, Scottish Intercollegiate Guidelines Network (1997) *Colorectal Cancer*. SIGN: Edinburgh

Scottish Cancer Therapy Network, Scottish Intercollegiate Guidelines Network (1998*a*) *Lung Cancer*. SIGN: Edinburgh

Scottish Cancer Therapy Network, Scottish Intercollegiate Guidelines Network (1998*b*) *Breast Cancer*. SIGN: Edinburgh

Scottish Intercollegiate Guidelines Network (1995) *Clinical Guidelines*: *Criteria for Appraisal for National Use*. SIGN: Edinburgh

Smyth JF, Mossman J, Hall R, Hepburn S, Pinkerton R, Richards M, Thatcher N, Box J on behalf of the United Kingdom Co-ordinating Committee on Cancer Research (1994) Conducting clinical research in the new NHS: the model of cancer. *Br Med J* **309**: 457–461

Twelves CJ, Thomson CS, Young J and Gould A (1998*a*) Entry to clinical trials in breast cancer: the importance of specialist teams. *Eur J Cancer* **34**: 1004–1007

Twelves CJ, Thomson CS, Gould A, Dewar JA for the Scottish Breast Cancer Focus Group and the Scottish Cancer Therapy Network (1998*b*) Variation in the survival of women with breast cancer in Scotland. *Br J Cancer* **78**: 566–571

© Cancer Research Campaign 1999
